# Asymptomatic peritoneal carcinomatosis originating from benign cystic peritoneal mesothelioma

**DOI:** 10.3332/ecancer.2015.605

**Published:** 2015-12-08

**Authors:** S Iacoponi, J Calleja, G Hernandez, R Sainz de la Cuesta

**Affiliations:** Gynecologic Oncology Unit, Quiron University Hospital, Calle Diego de Velasquez 1, Madrid, Spain

**Keywords:** benign multicystic mesothelioma, carcinomatosis, ovarian cancer, peritoneal cancer

## Abstract

Benign multicystic mesothelioma is a rare tumour that originates from the abdominal peritoneum with a predisposition to the pelvic peritoneum. It typically affects women of reproductive age. There have been less than 200 cases of this rare neoplasia reported to date.

We present the case of a 35-year-old woman who was referred to our centre because of the detection of a peritoneal carcinomatosis during a gynaecological exam. A diagnostic laparoscopy was performed. The findings included multiple cysts appearing as ‘a bunch of grapes’ occupying the omentum. Biopsies were taken during the surgery and the results showed benign multicystic peritoneal mesothelioma.

Benign multicystic mesothelioma can simulate other conditions, such as malignant ovarian tumours or cystic lymphangioma. It is often diagnosed accidentally during surgery performed for another reason. The diagnosis is interoperative, observing multicystic structures grouped as a ‘bunch of grapes’ containing clear fluid with thin walls made of connective tissue. Immunohistochemistry confirmed mesothelial origin. Surgery is considered the treatment of choice and is based on the removal of the cysts from the abdominal cavity.

Hyperthermic intraperitoneal chemotherapy can be considered as a primary treatment in patients with recurrences or even as a part of primary treatment associated with surgery. Survival at 5 years is 100% and invasive or malignant progression is extraordinary. The treatment approach should be multidisciplinary, and the patient should be referred to a referral centre.

## Introduction

Benign multicystic peritoneal mesothelioma (BMPM) is a rare tumour that originates from the abdominal peritoneum with a predisposition to the pelvic peritoneum. It typically affects women of reproductive age. There have been less than 200 cases of this rare neoplasia reported to date [1].

Due to the fact that it is a rare tumour, it is not well known and its aetiopathogenesis is not clear [2].

Occasionally, BMPM may present as an abdominal mass or even with obstructive symptoms, such as nausea, vomiting, or abdominal distension. For this reason, it can simulate malignant gynaecological tumours. Nevertheless, the diagnosis continues to be accidental in the context of a surgery performed for another reason or even in imaging tests requested for another purpose [3, 4].

Surgery is considered the treatment of choice and is based on the removal of the cysts from the abdominal cavity. BMPM is considered a benign entity; however, monitoring of these patients is fundamental due to the high rate of recurrence and the possibility, although rare or malignisation [5].

## Clinical case

**Figure 1.**
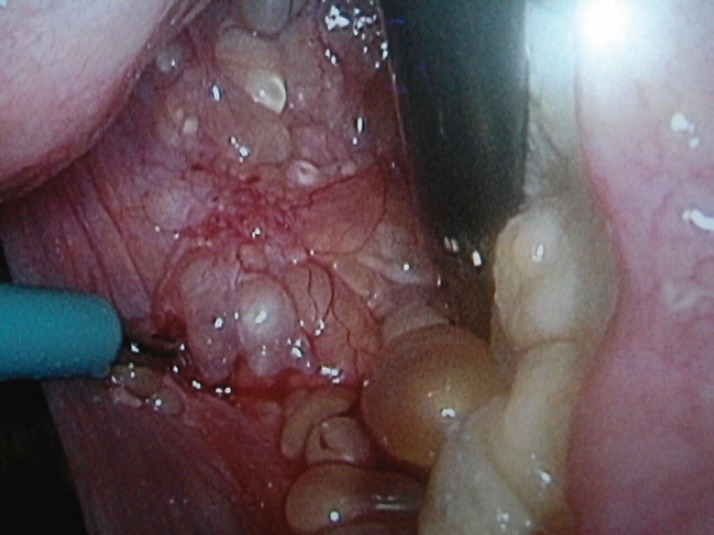
Laparoscopic image of implants in the Douglas pouch.

**Figure 2.**
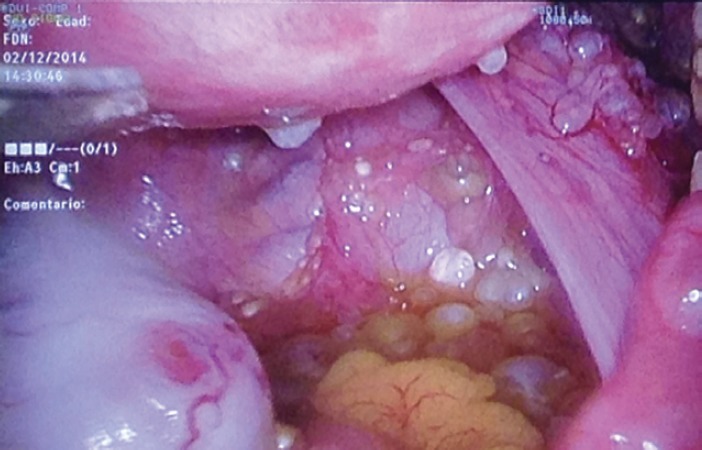
Laparoscopic image of multiple peritoneal implants in the Douglas pouch.

**Figure 3.**
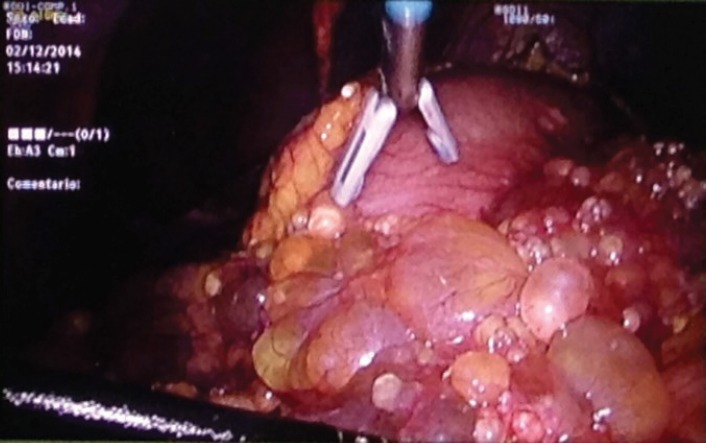
Laparoscopic image of multiple peritoneal implants at the level of the omentum.

Here, we present the clinical case of a 35-year-old woman who came to our centre as a referral from her gynaecologist following the detection of findings suggesting peritoneal carcinomatosis of probable gynaecological origin during a routine gynaecological examination. The patient had no personal or family history of interest, was asymptomatic, and the gynaecological examination was normal. The peritoneal markers were negative. The computerised axial tomography (CAT) scan and magnetic resonance imaging (MRI) showed several small hypodense peripheral lesions, which suggested mucinous implants at the level of the omentum, with attachment to the liver and between intestinal loops. Ascites with septations of probable mucinous origin were also observed. The appearance of internal genital structures showed no findings of interest. An exploratory laparoscopy was performed, in which multiple cysts in the form of ‘a bunch of grapes’ were observed occupying the omentum (Figures 1, 2 and 3). Multiple biopsies were taken during surgery for further study.

**Figure 4.**
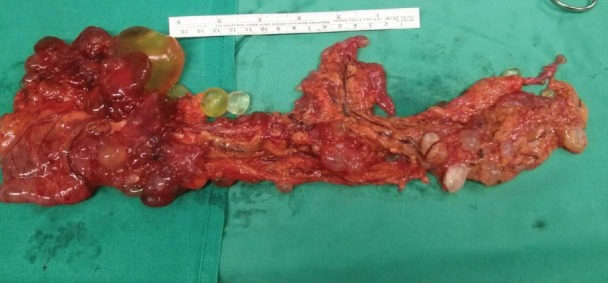
Surgical piece of the omentum.

The pathological anatomy indicates benign multicystic peritoneal mesothelioma. It was decided to convert the surgery to a laparotomy with omentectomy and ablation of all of the peritoneal implants without residual macroscopic tumours (Figure 4). After 1 year of monitoring, the patient is asymptomatic and free of illness at the present time.

## Discussion

BMPM can simulate other entities, including malignant tumours originating in the ovaries and cystic lymphangioma [6].

Abdominopelvic ultrasound, computerised tomography with contrast, or MRI may help, but in most cases the diagnosis can only be made during surgery. Although fine-needle aspiration can be used as a diagnostic tool, in most cases, this method does not allow us to reach a definitive diagnosis [7].

Laparoscopic exploration of the cavity is the most precise diagnostic method, as it permits the visualisation of the multicystic structures grouped into the typical ‘bunch of grapes’ formation and the taking of interoperative biopsies of the cysts, although it is still an invasive procedure. There is no consensus as to the recommended protocol in these cases [7, 8].

In our case, we chose diagnostic laparoscopy, as there was not a clear diagnosis using imaging techniques and a carcinomatosis was suspected.

The histological study shows vascularised cysts, full of transparent liquid, with thin walls made up of loose connective tissue. The immunohistochemical study demonstrates the mesothelial origin of the parietal cells, a fact that confirms the diagnosis [2]. The treatment is surgical and is based on the removal of the cysts [3].

Laparoscopy associated with laser therapy has been described as an alternative treatment for the destruction of the cysts, thus avoiding the risks and complications of multiple laparotomies, although there are few studies of this treatment [8].

Treatment with radiotherapy does not appear to be effective with these tumours [3].

A high rate of recurrence (27–75%) has been described, generally at the local level and in the long term [4]. For this reason, and due to the probability of malignisation, although until now only one case of malignant transformation of these tumours has been described in the literature [9], strict monitoring of these patients is required [5].

In the case of recurrence or even as part of the first-line treatment, some experts have proposed the combination of cyst reduction surgery with hyperthermic intraperitoneal chemotherapy (HIPEC) in selected patients [10].

The use of HIPEC along with surgery has demonstrated a low rate of recurrence (16.7%) in comparison to surgery along (41.7–50%). The prognosis is generally good with a mean survival at 5 years of 100% when the combined treatment is given [11].

Due to the fact that this is a rare tumour, there is little knowledge of its origin, behaviour, and response to treatment [12].

## Conclusion

The focus of the treatment for this disease should be multidisciplinary, studied by a medical committee in a specialised referral centre. At present, there are no validated recommendations for clinical management and no authorisation for sale in Europe of any cytotoxic agent indicated for treatment has been granted.

## Conflicts of interest

The authors declare that they do not have any financial or potential conflicts of interest of any kind.
